# The Relevance of the Endothelium in Cardiopulmonary Disorders

**DOI:** 10.3390/ijms25179260

**Published:** 2024-08-27

**Authors:** Laura de la Bastida-Casero, Bertha García-León, Olga Tura-Ceide, Eduardo Oliver

**Affiliations:** 1 Centro de Investigaciones Biológicas Margarita Salas (CIB), CSIC, 28040 Madrid, Spain; laura.bastida@cib.csic.es (L.d.l.B.-C.); bertha.garcia@cib.csic.es (B.G.-L.); 2 Translational Research Group on Cardiovascular Respiratory Diseases (CAREs), Dr. Josep Trueta University Hospital de Girona, Santa Caterina Hospital de Salt and Institut d’Investigació Biomèdica de Girona (IDIBGI-CERCA), 17190 Girona, Spain; otura@idibgi.org; 3 Department of Pulmonary Medicine, Servei de Pneumologia, Hospital Clínic-Institut d’Investigacions Biomèdiques August Pi I Sunyer (IDIBAPS), University of Barcelona, 08036 Barcelona, Spain; 4 Centro de Investigación Biomédica en Red Enfermedades Respiratorias (CIBERES), 28029 Madrid, Spain; 5 Centro Nacional de Investigaciones Cardiovasculares (CNIC), 28039 Madrid, Spain; 6 Centro de Investigación Biomédica en Red Enfermedades Cardiovasculares (CIBERCV), 28029 Madrid, Spain

**Keywords:** endothelium, cardiopulmonary disorders, pulmonary hypertension, chronic obstructive pulmonary distress, acute respiratory distress syndrome, COVID-19, therapeutic strategies

## Abstract

The endothelium is a cell monolayer that lines vessels and separates tissues from blood flow. Endothelial cells (ECs) have a multitude of functions, including regulating blood flow and systemic perfusion through changes in vessel diameter. When an injury occurs, the endothelium is affected by altering its functions and structure, which leads to endothelial dysfunction, a characteristic of many vascular diseases. Understanding the role that the endothelium plays in pulmonary vascular and cardiopulmonary diseases, and exploring new therapeutic strategies is of utmost importance to advance clinically. Currently, there are several treatments able to improve patients’ quality of life, however, none are effective nor curative. This review examines the critical role of the endothelium in the pulmonary vasculature, investigating the alterations that occur in ECs and their consequences for blood vessels and potential molecular targets to regulate its alterations. Additionally, we delve into promising non-pharmacological therapeutic strategies, such as exercise and diet. The significance of the endothelium in cardiopulmonary disorders is increasingly being recognized, making ECs a relevant target for novel therapies aimed at preserving their functional and structural integrity.

## 1. Introduction

The endothelium is a cellular layer that forms the luminal surface of blood vessels and plays an important role in vascular biology. The endothelium is made of endothelial cells (ECs) which have very important functions, including maintaining hemostatic balance to prevent thrombosis, regulate coagulation and vascular tone, and participate in the creation of new vessels. However, when an alteration or damage occurs in the endothelium, the endothelium becomes dysfunctional. This dysfunction is characterized by changes in the functions of ECs towards reduced vasodilation and creates a pro-inflammatory and pro-thrombotic state. These changes are associated with certain diseases such as hypertension, chronic heart failure, or viral infections, among many others that are discussed in this review [1].

The relevance of studying the role of the endothelium in cardiopulmonary disorders has increased considerably in recent years, as it has become a relevant target for potential new therapies [2], which are also reviewed in the present issue.

For this review, we searched in the PubMed and Google Scholar databases, merging both basic research on endothelium and clinical research on endothelial dysfunction and cardiopulmonary disorders. The terms used for the search were endothelium, endothelial dysfunction, COPD, PH, OSA, ARDS, and COVID-19 in combination with therapeutic strategies both pharmacological and non-pharmacological (exercise and nutritional approaches). Most of the articles used were recent research papers and reviews of the previous literature.

## 2. The Role of Endothelium in Cardiovascular and Pulmonary Vascular Physiology

The endothelium is a single layer of cells in the inner layer of blood vessels. It is in constant contact with the blood flow throughout the entirety of the circulation and plays essential roles in the homeostasis of the organism [1,3,4].

ECs can modulate the vascular tone by balancing both vasodilation and vasoconstriction factors. ECs release vasoconstriction factors such as endothelin-1 (ET-1), prostaglandins, and other components of the RAS system like angiotensin II, related to the regulation of calcium levels and in consequence responsible for the contraction of vascular smooth muscle cells (vSMCs) surrounding vascular ECs. ET-1 has been demonstrated to be present in ECs, along with von Willebrand factor (vWF) and other molecules in vesicles called Weibel–Palade bodies. On the other hand, ECs are capable of synthesizing vasodilator factors such as nitric oxide, prostacyclin, and c-natriuretic peptide, among others related to endothelium’s anti-thrombotic properties [1,3,5].

It is essential that in homeostasis, these processes are completely regulated. In this context, the endothelium usually has an anti-thrombotic and anti-aggregation state, which is arrested only if there is vascular damage. Only when an injury occurs, the endothelium induces platelet aggregation and leukocyte recruitment to the injury. In this case, platelet P-selectin creates a bridge between ECs, platelets, and leukocytes, forming aggregates within the injury [1]. The glycocalyx, a complex and fragile structure formed by proteoglycans and glycoproteins bound to plasma proteins like albumin and antithrombin present in the surface of ECs, regulates inflammation and vascular permeability among other functions [5].

The crosstalk between ECs and SMCs is necessary to adequately regulate the vascular tone. It has been reported that vascular ECs develop finger-like projection promoting physical contact between these two cell types, which could be related to calcium level regulation [3]. Moreover, it has been reported that EC-derived NO induces relaxation in SMCs after triggering the activation of soluble guanylyl cyclase (sGC) in adjacent smooth muscle, initiating in the latter cell type a relaxation-prone expression cascade and opposing a pro-inflammatory state within the cell [3].

The endothelium is crucial in both vasculogenesis and angiogenesis. While the first of these occurs mainly during development, the latter happens in adulthood with special relevance during repairment after injuries and certain pathologies. Angiogenesis requires vascular endothelial growth factor (VEGF) or other pro-angiogenic factors such as IL-8. These factors are responsible for initiating a cascade where cells migrate from the vessel sprout towards a gradient of VEGF. The migration occurs until another vessel sprout is found, thus generating a new vessel with blood flow, in a process called anastomosis [6].

Moreover, mitochondria are essential for EC homeostasis. PGC-1a, an essential regulator of mitochondrial function induces VEGF expression and angiogenesis, enhancing mitochondrial anti-oxidant defense [7]. VEGF, in turn, upregulates some mitochondrial genes that allow mitochondria to maintain an oxidative metabolism, using glucose, fatty acids, and amino acids in conditions of reduced glycolytic rates, thus helping ECs to survive in regions deprived of glucose [8].

The importance of the vascular endothelium is intrinsically relevant in the cardiopulmonary system. The pulmonary circulation retains all blood that has circulated through the systemic circulation where deoxygenated blood moves to the pulmonary arteries and into capillaries. It is in the pulmonary capillaries where gas exchange occurs in order to reoxygenate the blood so it can reenter the systemic circulation. ECs are therefore in close contact with the blood throughout the entire circuit [9]. Novel studies have highlighted the heterogeneity of lung endothelial cells with different physiological functions. Capillary endothelial cells are closer to alveoli, and therefore gas exchange, forming a more restrictive barrier to water and solutes compared with pulmonary arterial ECs. With scRNAseq assays, it has been possible to shed light on the heterogeneity of lung ECs. It is notable that these subtypes of cells have differential ion channel expression and permeability to solutes. In consequence, the metabolism and pro-inflammatory abilities of EC subtypes are different [10,11].

A correct balance of all the above-mentioned cellular and molecular pathways is essential to maintain endothelial homeostasis and, therefore, a healthy vascular function. Any alteration can cause a variety of vascular disorders, as we further explain as follows.

## 3. An Overview of the Most Relevant Cardiopulmonary Disorders

In this review, we highlight the importance of the endothelium within the pulmonary circulation and the cardiovascular system. In this regard, there are multiple diseases where both of these seem to be affected and where the endothelium plays an essential role in disease development. It is known that some of the hallmarks of the most relevant cardiopulmonary—or pulmonary vascular—disorders such as pulmonary hypertension (PH), chronic obstructive pulmonary disease (COPD), acute respiratory disease syndrome (ARDS), or obstructive sleep apnea (OSA) involve endothelial dysfunction. Some epidemiologic data such as their incidence, mortality, main symptoms, and identification in the general population are summarized in Table 1.

### 3.1. Pulmonary Hypertension (PH)

Pulmonary hypertension (PH) refers to a group of heterogeneous diseases associated with cardiopulmonary disorders affecting both the lungs and the cardiovascular system [26]. PH is a huge cause of mortality worldwide with different prevalence around the world, as it is a multifactorial disease [27]. The prevalence worldwide is around 1% and rises to 10% in elderly people (over 65 years) [12]. The main symptoms of this disease are dyspnea and exercise ability impairment, which can severely worsen quality of life [26]. The disease is characterized by high blood pressure and affects both heart and lung circulation. This is a consequence of the narrowing of the arteries, impairing the blood flow into these organs, causing hypertrophy of the right ventricle (RV), followed by RV failure and premature death [28,29].

PH can be classified into five broad groups. Group 1: Pulmonary arterial hypertension (PAH); idiopathic, hereditary, drug-induced, associated with tissue disease, HIV infection, congenital heart disease, portal hypertension, and persistent pulmonary hypertension of the newborn. Group 2: Pulmonary hypertension due to left heart disease; left ventricle (LV) systolic dysfunction, LV diastolic dysfunction, and congenital or acquired obstruction of the LV inlet or outlet tract. Group 3: Pulmonary hypertension due to lung disease and/or hypoxemia; chronic obstructive pulmonary disease (COPD), sleep-disordered breathing, chronic exposure to high altitudes, and lung development anomalies. Group 4: Chronic thromboembolic pulmonary hypertension and other obstructions of the pulmonary arteries; intravascular tumors, congenital stenosis of the pulmonary arteries, and parasitosis. Group 5: Pulmonary hypertension of an unestablished and/or multifactorial mechanism; hemolytic anemia, systemic diseases, and metabolic disorders [30,31].

PH can be pre-capillary PH, caused by an increase in the pulmonary artery pressure (PAP) and vascular remodeling of the pulmonary artery, presenting a pulmonary artery wedge pressure (PAWP) < 15 mmHg, including groups 1, 3, 4, and 5. Additionally, left heart disease (LHD) leads to elevated left atrial filling pressure. This causes reflux in the pulmonary veins and consequently increases the mPAP, known as isolated postcapillary hypertension (IpCPH) with a PAWP > 15 mmHg, included in Group 2 and some forms of Group 5. When IpCPH continues to progress, vascular remodeling occurs, increasing pulmonary vascular resistance (PVR) and inducing hypertrophy of the pulmonary vascular walls. This is known as combined PH, which is the simultaneous presence of both pre-capillary and post-capillary PH. Combined PH can be understood as an advanced IpCPH, less common and associated with a worse prognosis in patients [15,30,32].

However, it is in Group 1, pulmonary arterial hypertension (PAH), where the endothelium has a major role, as it is first affected and its dysfunction is essential for the onset of the disease. PAH is a rare, chronic progressive disease characterized by vasoconstriction, abnormal cell proliferation, fibrosis, altered cellular metabolism, thrombosis, and inflammation, with severe alterations in the ECs. This dysfunctional endothelium produces structural and functional changes in the pulmonary vasculature that are closely related to changes in vascular tone and remodeling. These changes are responsible of the progressive narrowing of the vessel which causes an increase in PAP. PAH is characterized by increased mean pulmonary arterial pressure (mPAP) at rest > 20 mmHg. Subsequently, the continuous RV overload causes RV hypertrophy, which over the time provokes the loss of its contractile function, generating RV systolic dysfunction related to a worse prognosis. Currently, there are treatments that improve patients’ quality of life, but none achieve a total cure of the disease, which leads to right heart failure and consequently death [15,33,34]. Endothelial dysfunction has also been shown to be present in chronic thromboembolic pulmonary hypertension (CTEPH), classified as group 4 PH. Previous studies have demonstrated a hyperproliferative dysfunctional phenotype and abnormal metabolism in CTEPH endothelial cells, highlighting their potential contribution to the progression of unresolved thrombi and vascular remodeling [35,36,37].

### 3.2. Chronic Obstructive Pulmonary Disease (COPD)

Chronic obstructive pulmonary disease (COPD) is a chronic inflammatory disease of the lungs and peripheral airways, which limits airflow [38]. At the beginning of the disease, patients feel a shortage of breath when exercising, but as the disease progresses, they begin to have difficulty exhaling and inhaling [39]. Furthermore, there are two pathological processes caused by COPD: the remodeling and narrowing of the airways; and the destruction of the lung parenchyma, which causes the closure of the airways generating dyspnea in these patients, worsening when exerting effort [38]. In COPD, the endothelium is also altered, presenting endothelial dysfunction defined as inadequate vasodilation. This event results in disruption of the microvascular endothelial barrier and the loss of antiadhesive and antithrombotic functions of the endothelium. Studies have shown the presence of endothelial dysfunction in the pulmonary arteries of patients with early COPD, suggesting that this phenomenon occurs at the beginning of the disease. Furthermore, the presence of risk of cardiovascular disease has been related to patients with COPD who present higher levels of endothelial dysfunction. Therefore, the pathogenesis of COPD is closely related to endothelial dysfunction [40].

There are multiple risk factors, including air pollution, the presence of other pulmonary diseases such as asthma, genetics, and smoking [38,41]. Tobacco smoke contains tar that reaches the lungs, which causes damage to the organ. This event generates alterations in the ECs which begin to suffer oxidative stress, undergo apoptosis, and produce inflammatory mediators favoring an increase in T-lymphocyte infiltration that damages the lungs [42,43].

In the clinical course of the disease, patients frequently suffer exacerbations that are characterized by acute worsening of symptoms and bad prognosis, affecting the progression and control of the disease [39]. According to reports, 50–80% of patients who suffer from COPD die due to respiratory causes, from exacerbation of the disease (30–50%), lung neoplasia (8–13%), or due to other causes of respiratory origin [44].

### 3.3. Acute Respiratory Distress Syndrome (ARDS)

ARDS is a clinical syndrome of diffuse lung inflammation and non-cardiogenic edema that is usually caused by acute respiratory failure [45]. In this category, complications after infection with Severe Acute Respiratory Syndrome Coronavirus 2 (SARS-CoV-2), which caused the COVID-19 pandemic, can be included [46]. Although COVID-19 is mentioned within this context, infection by pathogens is not the only cause of ARDS. It can be consequence of indirect (such as non-pulmonary sepsis, ischaemia reperfusion after lung transplant, or drug toxicity) or direct lung injury (such as bacterial, viral, or fungal pneumonia, pulmonary contusion, or aspiration of gastric contents) [45,47]. The main characteristic of ARDS pathophysiology is excess inflammation that causes diffuse alveolar damage and hemorrhage resulting in edema fluid accumulating within the lungs.

The endothelium and its dysfunction play an important role in the pathogenesis of ARDS, as the increase of endothelial permeability allows the pulmonary edema to form. Recent studies have shown that patients post-COVID-19 infection exhibit an abnormal increase in endothelial progenitor cells as a result of endothelium damage [48,49]. Not only the endothelium but also epithelial permeability accounts for the breakage of the lung microvascular barrier via the destabilization of VE-cadherin and E-cadherin junctions, respectively, permitting immune cell leakage [45,50].

The presence of liquid within the lungs can be reabsorbed in a process called alveolar fluid clearance, where an osmotic gradient is formed to enable fluid removal. However, in ARDS and depending on the degree of the disease, alveolar fluid clearance is hindered, resulting in a poor prognosis [45].

In order to understand the pathophysiology of ARDS, it is relevant to acknowledge the physiology of the alveolar–capillary barrier, including how both epithelial and endothelial cells are injured when alveolar flooding happens because of the rupture of these both barriers. Moreover, the outcomes for patients suffering from ARDS are variable, although ARDS is associated with high mortality; in patients where the edema resolves, there is high risk of developing fibrosis that impedes the complete recovery of patients [45,50].

### 3.4. Obstructive Sleep Apnea (OSA)

OSA is a sleep-related breathing disorder where obstruction of the upper airways causes episodes of apnea or hypoapnea, depending whether the breathing cessation is complete or only partial, therefore causing intermittent hypoxia (IH). The degree of severity of the disease depends on the number of hypoxic events that occur [25]. It is relevant to mention that the presence of OSA can increase the prevalence of other cardiovascular and pulmonary diseases, such as atherosclerosis or myocardial infarction [51].

OSA is associated with an impaired endothelial function that is currently not well understood but seems to be related to different pathways connected with the state of intermittent hypoxia [52]. Excess oxidative stress with an increased synthesis of reactive oxygen species (ROS) and reactive nitrogen species (RNS) also impairs vasodilation as there is less NO available. It is known that the source of ROS and RNS is the mitochondria, and the stress induced in mitochondria after OSA tends to cause mitochondrial dysfunction [25]. The decreased availability of NO involves asymmetric dimethylarginine (ADMA) and monomethylated arginine (L-NMMA), creating competitive feedback inhibiting eNOS. It is relevant that increased levels of ADMA are associated with endothelial dysfunction [51].

Studies have shown that IH impairs endothelial function with a decreased proliferation of endothelial progenitor cells (EPCs) in the blood. Microvesicles secreted by EPCs during IH have been associated with pro-apoptosis and pro-oxidative processes, in line with an impaired endothelial cell function [51,53].

Interestingly, inflammation is also increased in OSA patients, not only causing systemic inflammation that is related to a chronic inflammation usually seen in cardiovascular diseases but also airway inflammation due to mechanical stress related to snoring-induced airway vibration [25]. An increased presence of markers such as C-reactive protein (CRP), leptin, interleukins IL-6, IL-8, and tumor necrosis factor α (TNF-α) were observed in this disease [54].

The occurrence of IH in OSA induces a sympathetic activation that exacerbates the vasoconstriction, which is affected by the decreased vasodilation caused by the lower presence of NO. All of this leads to a higher risk of developing different vascular diseases such as coronary artery disease, stroke, or peripheral artery disease, which are the main causes of morbidity in OSA [25].

The mechanisms that drive endothelial dysfunction in OSA are mainly driven by impaired production of NO via eNOS disruption, an increased inflammatory response within the endothelium, and enhanced apoptosis.

### 3.5. Air Pollution

Whilst air pollution cannot be considered a disease, it is relevant to the development or worsening of some of the cardiopulmonary disorders mentioned already [55]. Air pollution comprises solid and/or liquid particles suspended in the air. These particles have various sizes; the most relevant ones to respiratory diseases are particulate matter (PM)2.5 and PM10, smaller than 2.5 or 10 µm, respectively [56].

Exposure to different air pollutants can impair endothelial function with augmented levels of inflammation (with increased levels of TNF-α, CRP, ET-1, IL-1β). Interestingly, some studies report that exposure to PM2.5 increases the levels of EC apoptosis [56].

Even though the general mechanisms of endothelial pathologies are similar, there are specific biomarkers found in each of the above-mentioned diseases that can help us to better understand them (Table 2). Some of the most relevant pathological pathways are further analyzed in the next section of this review.

## 4. The Pathological Mechanisms of Pulmonary Endothelial Dysfunction

**Figure 1:**
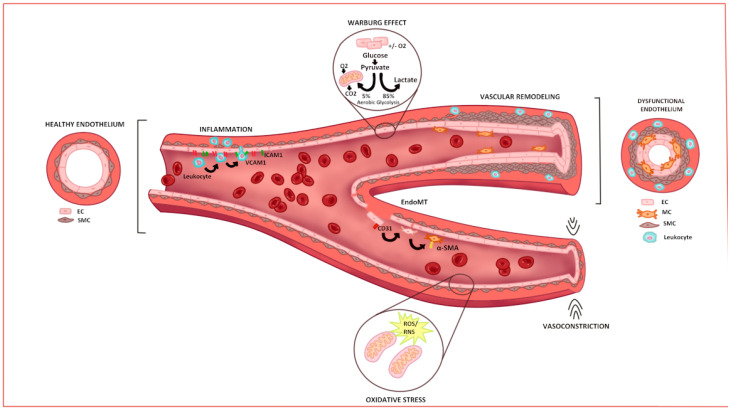
Main mechanisms of pathology in endothelium. Blood vessels are made up mainly of ECs and SMCs, which line the inside of the vessels, also covered by a layer of fibroblast that confers structure. After damage, multiple processes occur such as inflammation, increased oxidative stress, increased aerobic glycolysis in ECs, the transformation of ECs to MCs, vasoconstriction, and finally, vascular remodeling due to the proliferation of ECs and SMCs. All of this generates dysfunction of the endothelium, which reduces the amount of blood flow that can circulate through the vessels. This dysfunction is characteristic of the diseases discussed in this review. CO_2_: carbon dioxide; EC: endothelial cell; EndMT: endothelial mesenchymal transition; ICAM-1: intercellular adhesion molecule 1; MC: mesenchymal cell; RNS: reactive nitrogen species; ROS: reactive oxygen species; O_2_: oxygen; SMC: smooth muscle cell; VCAM-1: vascular cell adhesion molecule 1.

Pulmonary artery EC dysfunction is characterized by an imbalance between vasoconstrictor excess (ET-1 pathway) and a lack of vasodilators (NO and prostacyclin pathways), leading to vasoconstriction and proliferation [58]. These alterations of the endothelial layer are also related to metabolic alterations and oxidative stress, and can induce inflammation and endothelial-to-mesenchymal transition (EndoMT), among other phenomena that are briefly summarized in Figure 1.

### 4.1. Inflammation

Tobacco smoke, pollution, or even the presence of diseases produce small injuries in the endothelium, which becomes more permeable to different molecules [41]. In this way, many circulating lipoproteins enter into the intima layer through the endothelium. These lipoproteins, together with inflammation and oxidative stress, induce the production of cytokines such as TNF-α, which is liberated by immune cells. TNF-α binds to its receptor TNFR, causing inflammation, apoptosis, ROS, cell proliferation, and cell survival [59]. The binding of TNF-α to TNFR induces the expression of adhesion molecules, including intercellular adhesion molecule 1 (ICAM-1) and vascular cell adhesion molecule 1 (VCAM-1), in the endothelium [59]. This allows circulating leukocytes to adhere to the damaged endothelium. Inflammation leads to a weakening of the junctions between endothelial cells, generating transendothelial migration of leukocytes to the intima layer where they accumulate and liberate large amounts of neutrophil elastase (NE) and ROS, destroying the alveolar epithelium [41,59,60]. In diseases such as PAH, the hormone leptin is excessively produced and liberated, similar to a cytokine, acting as a key modulator of regulatory T-cell hyporeactivity [58].

### 4.2. Oxidative Stress

Under physiological conditions, oxidative stress is produced in the mitochondrial respiratory chain. However, there are pathological processes that generate excessive oxidative stress, such as inflammation. The excess increase in ROS generates damage to biomolecules such as nucleic acids, proteins, and lipids, which leads to cell death. This event is what happens in cardiovascular diseases, where there is an increase in ROS and a decrease in antioxidants [1,5,8].

The main mechanism by which oxidative stress occurs in ECs is the inactivation of NO by superoxide anions. These free radicals inhibit the activation of endothelial receptors for acetylcholine, serotonin, thrombin, and many other mediators, generating a decrease in NOS activity in ECs. In this way, the production of NO is reduced, which leads to vasoconstriction and the formation of atherosclerotic plaque. Furthermore, ROS production can react with NO and generate peroxynitrite anion (ONOO-) and nitrogen dioxide, leading to inflammatory injury to the vasculature and decreased NO availability [1,61]. NO controls hydrogen sulfur (H2S) levels by inhibiting or stimulating the activity of the enzyme cystathionine γ-lyase (CSE), which is responsible for H2S production. H2S is produced in ECs, where it has a very important role participating in the inhibition of vascular inflammation. Opening ATP-sensitive K^+^ channels in SMCs which generates vasorelaxation, it positively regulates antioxidant enzymes such as catalase or superoxide dismutase (SOD), reducing ROS levels in cells, inhibits the proliferation of SMCs, and stimulates the proliferation of ECs, promoting angiogenesis. In pathological situations where there is endothelial dysfunction, lower H2S availability occurs due to decreased gene expression of H2S generating enzymes in the endothelium [62]. In the study carried out by Yan and his collaborators in 2004 [63], they observed a reduction in the bioavailability of H2S in hypertensive rats. In fact, it was predicted that when H2S decreased in rats, an increase in blood pressure occurred, demonstrating a negative correlation between H2S bioavailability and blood pressure.

Factors such as smoking, hypertension, and other respiratory diseases cause EC damage by increasing the presence of ROS, which contributes to the endothelial dysfunction characteristic of these diseases [1,61].

For example, patients with COPD are not able to reduce the accumulation of ROS, because they have a lower level of nuclear factor E2 (Nrf2) in cells, which is responsible for regulating antioxidant genes. When there is an increase in oxidative stress, this factor is transported to the nucleus to activate the transcription of these genes. However, COPD patients have lower endogenous production of antioxidant genes as a consequence of low levels of Nrf2 [60].

Moreover, ROS increases with the stack of neutrophils and the liberation of IL6, destroying the lung tissue structure. In addition, neutrophils secrete serine proteases such as matrix metalloproteinase (MMP) and neutrophil elastase (NE). MMP destroys the extracellular matrix of the lung, generating remodeling of the airways. Furthermore, the neutrophils’ aggregation leads to the activation of inflammatory factors that produce even more ROS, aggravating the response to oxidative stress. The increase in ROS decreases the activity of histone deacetylases (HDACs), leading to greater aggregation of inflammatory cells (mainly neutrophils) [60].

### 4.3. Vascular Remodeling

The lung vasculature has to respond to rapid variations in pressure, shear stress, and vascular resistance. However, when any of these factors are excessive, it can lead to vascular remodeling. It has been extensively reported that vascular remodeling happens in some cardiopulmonary disorders such as PH and COPD [46]. Vascular remodeling is characterized by thickening of the arterial wall, with reduced vessel lumen that inevitably leads to increased pulmonary vascular resistance.

At the beginning of the disease, ECs apoptosis is observed; however, as the disease progresses, ECs hyperproliferate and become resistant to apoptosis, contributing to remodeling of the vessels. In this way, hyperplasia and proliferation of SMCs occur, which also contribute to the remodeling and the recruitment of inflammatory cells [33].

In response to inflammatory mediators (IL-1, TNF-α) that are released within the organ from activated monocytes, ECs as well as SMCs secrete growth factors that enhance atherogenesis. Furthermore, ET-1 is a powerful activator of mitosis of SMCs, which generates their proliferation and consequently pulmonary vascular remodeling. Elevated levels of ET-1 were observed in the lungs and plasma of patients with PAH [64].

It is notable that in other pulmonary disorders such as COVID-19-induced ARDS the level of vascular remodeling is even higher than in PH with thrombosis, endothelitis, and vascular inflammation. This excessive remodeling seems to be mainly a consequence of hypoxia due to decreased blood flow associated in COVID-19 with the presence of thrombus [46], in a similar manner that occurs in Group 1 and 4 PH [65]. Both groups show a dysregulated hyperproliferation in intima, media, and adventitia layers, resulting in an aberrant vascular remodeling and narrowing of the blood vessels, thus enhancing blood pressure. Moreover, Group 4 (CTEPH) also begins with the formation of thrombus, thus sharing similarities with COVID-19.

### 4.4. Endothelial-Mesenchymal Transition (EndMT)

EC can contribute to arterial remodeling through endothelial mesenchymal transition (EndMT). EndMT is a process in which ECs lose endothelial properties and acquire those of a mesenchymal cell. Firstly, ECs lose their cell–cell junctions via endothelial transforming growth factor beta (TGFβ) signaling, degrading the basement membrane and migrating out into the perivascular surroundings [66,67]. TGFβ is also known to induce EndMT via epigenetic reconfiguration by regulation of transcription factors commonly associated with EndMT. This occurs either through regulation of EndMT transcription factors such as Snail1 or SMAD molecules. New advances have associated miRNA-mediated modulation with TGFβ-induced EndMT [66]. When loss of the integrity of the endothelium barrier occurs, cellular signaling begins to alter, which generates a vasoconstrictor and vasodilator imbalance [33].

EndMT is characteristic by many pathological processes. It is known that hypoxia, inflammation, and oxidative stress are factors that contribute to EndMT, and that all these factors are present in PH and PAH. However, although hypoxia occurs in OSA, it is not yet known whether this phenomenon occurs in this disease. The same applies for ARDS patients, since in one study, treatments aimed at reducing EndMT in sick patients showed a clinical improvement; however, the phenomenon of EndMT in this disease remains poorly known and more research is needed. Furthermore, in a study with ARDS in mice, it was shown that obesity induced by a high-fat diet reduced endothelial alteration by suppressing EndMT, as it reduced fibroproliferation, lung hyperpermeability, and oxidative stress [68,69,70]. In addition, it has been seen that epithelial–mesenchymal transition (EMT) occurs in smokers with COPD, a process in which epithelial cells lose polarity and transform into mesenchymal cells. EMT has been shown to be related to the increase of fibroblast-specific protein 1 (FSP-1). In this way, there are indications that just as EMT is activated, EndMT could be activated in these patients too. In fact, in the study carried out by Sohal in 2016, the cells stained positive for FSP-1 were thought to be ECs in transition; in this way, the EndMT process is active in patients with COPD, contributing to pulmonary vascular remodeling [71].

### 4.5. Metabolic Dysfunction

The pulmonary endothelium has a very active metabolism as it has to rapidly adapt to environmental changes; therefore, mitochondria are essential organelles in the lung endothelial cells [72]. Glucose is the main source of energy in ECs, similar to most tissues. This is why glycolysis is an essential process in ECs. Pyruvate formed during glycolysis can enter the tricarboxylic acid (TCA) cycle in order to produce ATP, which fuels ECs. Fatty acids are also a relevant source of energy for lung ECs after undergoing fatty acid oxidation, relating to the carnitine palmitoyl transferase (CPT) system as well as glutamine metabolism [72,73].

However, it has been reported that glycolysis is enhanced in ECs even in a healthy scenario, in order to save oxygen for the diffusion of oxygen [74]. Interestingly, the glycolysis rate in lung ECs is much greater than in other cell types in other tissues, even resembling the rate of glucose consumption in cancer cells. This is what is called the Warburg effect, which is known in numerous human cancers and consists of greater consumption of glucose by cells for the synthesis of ATP, even in the presence of hyperoxia [58].

The inflammatory response that occurs during disease that affects ECs synergizes the change to a glycolytic metabolism. There is positive regulation of pyruvate dehydrogenase kinase (PDK), which inhibits pyruvate dehydrogenase (PDH), so it cannot induce the oxidative decarboxylation of pyruvate, which leads to a decrease in energy production by ECs and mitochondrial ROS production. Therefore, it translates into mitochondrial dysfunction from ECs. Furthermore, the activation of several proteins that generate cell proliferation occurs because of negative regulation of miR-124 in diseased ECs. MiR-124 binds to polypyrimidine tract binding protein 1 (PTBP1), which is responsible for regulating the glycolysis flux. When PTBP1 is not inhibited by MiR-124, it produces an increase in glycolysis, which results in hyperproliferation of ECs. In addition, diseased ECs overexpress fatty acid synthase (FASN), which is a multienzyme complex that catalyzes fatty acid biosynthesis. Overexpression of FASN leads to increased proliferation, since inhibition or silencing of FASN expression generates apoptosis and autophagy, decreasing the proliferation of ECs [8].

Interestingly, the glycolysis rate in diseased tissue can increase more, which is related to higher levels of VEGF that induce excessive angiogenesis, generating dysfunctional blood vessels. Vasoprotection is also affected due to a decrease in nitric oxide (NO) production because of decompensation of endothelial nitric oxide synthase (eNOS) [74].

One of the hallmarks of PAH is the presence of plexiform lesions where endothelial cells do not maintain a monolayer of cells; these cells present pro-proliferative and anti-apoptotic characteristics which are in close association with their metabolism. PAH ECs present increased glycolysis as well as the pentose–phosphate pathway (PPP). The latter mediates the formation of NADPH intermediates, resulting in the production of ROS [72].

### 4.6. Angiogenesis

Angiogenesis is a process that consists of the growth of new blood vessels. This is related to vasculogenesis, which is based on the development of vascular structures using endothelial progenitor cells. Angiogenesis is a very important process for wound healing, tissue development, and embryonic growth. The angiogenesis process occurs through mediators that stimulate vessel growth, generating stable and functional vessel networks. Among the angiogenic mediators we find VEGF, fibroblast growth factor (FGF), angiopoietin, platelet-derived growth factor (PDGF), monocyte chemoattractant protein-1 (MCP-1), integrins, VE-cadherin, and NO. In pathological conditions in which inflammation occurs, an increase in VEGF has been found, which generates excessive angiogenesis and an increase in vascular permeability, causing an increase in molecules such as ICAM, VCAM, and fatty acids, among others. Therefore, in pathological situations, aberrant angiogenesis occurs, which can help the development and progression of multiple diseases such as cancer and cardiovascular disease [75].

One of the main characteristics of COPD is the presence of remodeled small airways caused by an aberrant induction of angiogenesis. The exacerbated inflammation caused in COPD induces overexpression of angiogenesis mediators such as VEGF, basic fibroblast growth factor (B-FGF), and TNF-α. Some studies have correlated the presence of endothelial dysfunction as an initiating event for angiogenesis, as the endothelium regulates cell growth in the vessel wall [76].

### 4.7. Vasoconstriction

As we have already mentioned, dysfunction in the endothelium generates a decrease in vasodilator molecules such as NO and prostacyclins, and an increase in vasoconstrictor molecules such as ET-1. Prostaglandin I2 is generated in the endothelium and is a pulmonary vasodilator that acts on SMCs producing vasodilation through an increase in cAMP. In addition, it inhibits the proliferation of SMCs and platelet aggregation. It has been seen that in the ECs of patients with PAH, prostaglandins and NO are decreased. Additionally, ET-1 is a peptide produced in the endothelium that has two types of receptors: endothelin A (ETRA) and endothelin B (ETB), which are found in the SMCs of the pulmonary arteries. When ET-1 binds to ETRA, intracellular Ca^2+^ concentrations increase, activating protein kinase C (PKC) and contributing to vasoconstriction of the arteries [64].

## 5. Current Therapeutic Strategies in Pulmonary Vascular Diseases

Currently, there are treatments that manage to improve the lives of patients who suffer from PH. However, the vast majority try to replace the damaged endothelial function or avoid its pathological consequences rather than restore or protect ECs. In this context, it is remarkable that these treatments have been approved only for PH and that little is known about their potential impact in others among the above-mentioned pulmonary vascular diseases like ARDS, COPD, or OSA. Some of the most common treatments for PH are calcium channel blockers (CCBs), endothelin receptor antagonists (ERA), inhibitors of phosphodiesterase type 5 (IPDE5), and stimulators of soluble guanylate cyclase (SGCSs), as well as prostacyclin analogues (PA), prostacyclin receptor agonists (PRAs), and Sotatercept.

### 5.1. Calcium Channel Blockers (CCBs)

ECs and SMCs are connected through autocrine, paracrine, and endocrine signals controlled by Ca^2+^ that modulate different functions such as vasodilation. The different concentrations of Ca^2+^ cause the SMCs to be contracted or relaxed. When the endothelium is dysfunctional, the ECs begin the signaling cascade that ends up increasing Ca^2+^ concentration in the SMCs, thus inducing vasoconstriction. CCBs reduce intracellular Ca^2+^, preventing vasoconstriction [58,77,78]. Some of them include nifedipine, diltiazem, and amlodipine at high doses, administered at progressive doses over time [30]. However, they are not used frequently, as they can lead to hemodynamic compromise [58].

### 5.2. Endothelin Receptor Antagonists (ERA)

ET-1 is synthesized in the ECs and acts in the SMCs of the vasculature, where it binds to its two receptors (ETAR and ETBR) that are coupled to Gq protein forming inositol triphosphate (IP3). On the one hand, when ET-1 binds to ETAR, it generates an increase in IP3. Calcium is liberated by the sarcoplasmic reticulum, causing vasoconstriction of the SMCs at the same time as cell proliferation, tissue fibrosis, and endothelial damage, which results in diseases such as PH. On the other hand, the binding of ET-1 to ETBR generates the production of NO by ECs, which relaxes the vascular smooth muscle, thus inhibiting vasoconstriction and cell proliferation. For this reason, ETAR inhibitors are used as a possible treatment for diseases such as PH to reduce vasoconstriction. Some of the most commonly used ERAs are ambrisentan, bosentan, and macitentan, which improve pulmonary hemodynamics. Bosentan competitively antagonizes the binding of ET-1 to the ETA receptors and irreversibly blocks their activities [30,58,78,79].

### 5.3. Inhibitors of Phosphodiesterase Type 5 (iPDE5) and Stimulators of Soluble Guanylate Cyclase (sGC)

Under healthy conditions in the vascular endothelium, the NO produced by eNOS reaches the SMCs. NO activates the sGC which, through GTP, produces cyclic guanosine monophosphate (cGMP). This induces relaxation and antiproliferation in the vascular smooth muscle. iPDE5 inhibits cGMP catabolism and sGC promotes its synthesis from GMP. Some iPDE5 used are sildenafil and tadalafil, which have been shown to improve symptoms, exercise capacity, and pulmonary hemodynamics in PAH [30]. Furthermore, one of the most sGC activators used, riociguat, improves the synthesis of cGMP even in the absence of NO, so it is independent of endogenous NO. In addition, it stabilizes the bond between NO and sGC by increasing cGMP. Riociguat also improves symptoms and pulmonary hemodynamics; however, it must be used carefully since in combination with iPDE5 it can cause syncope, so both medications should never be combined [30,78].

### 5.4. Prostacyclin Analogues (PA) and Prostacyclin Receptor Agonists (PRA)

Prostacyclin is a potent vasodilator and antiplatelet agent with antiproliferative and antithrombotic properties [30]. Prostanoids are liberated by EC from the pulmonary artery and bind to their receptors on SMCs. The binding with its receptor generates the activation of adenylate cyclase, which produces an increase in cAMP. This results in vasodilation. In addition, intracellular prostacyclins bind to PPAR nuclear receptors on the SMCs, inhibiting the kinase responsible for activating genes for SMC proliferation. In general, patients with diseases such as PAH have little prostacyclin production [58]. Therefore, these patients are administered epoprostenol, which is a synthetic prostacyclin. Epoprostenol has two mechanisms of action; it acts as a vasodilator agent of the pulmonary vascular artery, and inhibits platelet aggregation. However, its use presents complications and a series of serious side effects such as metabolic alterations including hyperglycemia or hyperthyroidism [30,58].

### 5.5. Sotatercept

In PH, a deregulation of the Smad1/5/8 pathway of the bone morphogenic protein (BMP) type II receptor occurs in SMCs and ECs. Downregulation of BMPR-II-Smad1/5/8 increases the production of Activin A and GDF11, which increases the activity of Smad2/3, promoting the expression of endogenous BMP antagonists. The result is that antiproliferative signaling is reduced, shifting the balance toward cell proliferation, resulting in pulmonary vascular remodeling. Sotatercept is an activin receptor IIA-Fc (ActRIIA-Fc) fusion protein, which acts by trapping activin A ligands and GDF11. Its mechanism of action consists of maintaining a balance between proliferative and antiproliferative signaling pathways [80]. In animal models with PH, it has been seen that Sotatercept inhibits cell proliferation, activates apoptosis, and reduces inflammation in the vessels, restoring their permeability. Although more studies are required to better understand its clinical potential, it was observed that PAH patients treated with Sotatercept in a clinical trial had improved hemodynamic parameters [81].

As we have mentioned, none of these strategies have a direct impact on the endothelium. There is, therefore, a need to finding new targets to protect and regulate the unhealthy endothelium, able to prevent the fatal consequences of endothelial dysfunction.

## 6. New Potential Therapeutic Targets Focus on the Endothelium

There are possible new candidates that could be therapeutic targets for the above-mentioned cardiopulmonary disorders in which the endothelium is damaged and dysfunctional. Some recent studies in which omics techniques and single-cell sequencing—allow researchers to understand cell heterogeneity—have been performed, showing genes and signaling pathways that are altered in the ECs of diseased mice [33,82]. For example, in the study carried out by Rodor et al., 2022, around 222 genes showed differences between PAH and the control group. Out of those 222, 14 showed statistically significant differences between PAH ECs and controls (CD74, H2-Ab1, H2-Aa, H2-Eb1, Sparcl1, QSOX1, Tap1, H2-DMa, CD34, H2-DMb1, Aqp1, Milpda, Plvap, and Abi3bp) [33]. In this review, we have therefore selected the following genes as strong candidates to be new potential targets to treat endothelial damage-related diseases.

### 6.1. Sgk1

This gene encodes serum and glucocorticoid-induced kinase 1 (SGK1), which is a serine–threonine kinase that plays an important role in the cellular stress response. It activates certain sodium, potassium, and chloride channels, participating in regulating processes such as cell survival and renal sodium excretion [83]. SGK1 is genomically upregulated by mineralocorticoids and TGF-B. Furthermore, the nuclear inflammatory transcription factor κB (NFκB) is upregulated by SGK1, which stimulates the expression of various inflammatory mediators [84].

Overexpression of SGK1 is observed in a wide variety of fibrosing diseases including pulmonary fibrosis, obstructive kidney disease, obstructive nephropathy, liver cirrhosis, Crohn’s disease, and celiac disease. The adverse inflammatory and fibrosing effects of mineralocorticoids can be mitigated or even reversed by mineralocorticoid receptor blockers, so they can be considered in the treatment of inflammatory and/or fibrosing diseases [84].

Furthermore, ECs have the ability to migrate to expand and reach different tissues, supplying enough oxygen and nutrients depending on metabolic demands [74]. The SGK1 gene is also responsible for regulating angiogenesis and its deficiency has been shown to prevent PAH, as it is a key regulator of the primary changes that occur in PAH ECs. In a previous study carried out by Rodor et al., 2022, it was seen that this gene was positively regulated in the arterioles and vessels of mice with PAH. The vasculature is affected by remodeling and muscularization in this disease, so it could become a good candidate as a therapeutic target [33]. Furthermore, in another study carried out by Sierra et al., 2020, they demonstrated that inadequate SGK1 activity represented a risk factor in the development of metabolic syndrome with hypertension [83].

### 6.2. Sparcl1

Secreted protein acidic and rich in cysteine-like protein 1 (SPARCL1) is an extracellular matrix protein that inhibits angiogenesis and shows high expression after endothelial injury. SPARCL1 induces a pro-inflammatory phenotype by increasing cytokine levels like IL6, TNF, IL4, IL10, and IFN-γ through the activation of TLR4 transmembrane protein localized in macrophages. This exacerbates inflammation and increases the severity of the disease by inducing the polarization of macrophages to the M1 subtype that is widely known to exacerbate inflammation and decreasing the M2 subtype, which is anti-inflammatory [85]. In addition, it has been reported that it contributes to angiogenesis in PAH and is a biomarker of remodeling in the RV as it plays an important role in the regulation of fibrosis, with overexpression found in experimental models of cardiac hypertrophy and fibrosis [33,86].

In a previous study carried out by Zhao and his collaborators in 2024, it was demonstrated how lung capillary ECs presented altered transcriptomics and adopted a different phenotype after viral injury, such as COVID-19, to generate high levels of SPARCL1. In fact, circulating SPARCL1 was higher in patients who died from COVID-19, unlike recovered sick patients, where the levels of this protein were much lower. This shows the great potential that this protein has as a possible biomarker for patient prognosis. In addition, it was shown that mice with genetic ablation for SPARCL1 (ECSparcl1-KO) presented mild symptoms of pneumonia, less loss of bodyweight, and faster recovery with improving oxygen saturation compared with WT mice. Also, ECSparcl1-KO mice had a higher probability of survival compared with WT, and although no significant differences were observed between groups, there was a downward trend in inflammatory cytokines (TNF-α and IL-6). The study showed that the endothelial deletion of this protein also affected the number of active macrophages; however, the same did not occur with the rest of the immune cells. Furthermore, SPARCL1 deficiency protects against pneumonia due to COVID19, as these mice were able to attenuate local inflammation. Similarly, it was also shown that overexpression of SPARCL1 generated the opposite effects, worsening pneumonia in animals [85].

### 6.3. CD34

CD34 is a transmembrane protein located in ECs that is strongly expressed in large blood vessels, such as arteries and veins, as well as in smaller capillaries. In pathologies, the endothelial expression of CD34 is modified, so it has been proposed as a marker of disease progression [87]. On the other hand, CD34 is a glycophosphoprotein that is expressed in hematopoietic stem cells and in cells of different tissues, more specifically in the vessel wall. In addition, CD34^+^ cells have been seen to contribute to both endothelial regeneration and the inflammatory response [88].

In the study carried out by Jiang et al. in 2021, about 10% more CD34-expressing cells were found in diseased human arteries (with peripheral artery disease) compared with healthy ones. Furthermore, it was demonstrated that after injury to the endothelium, the ECs of the damaged artery regenerated from CD34^+^ cells, contributing to the formation of new microvessels in the injured artery [88].

### 6.4. MIF-CD74

Studies have demonstrated that CD74, a transmembrane glycoprotein that is the receptor for macrophage migration inhibitory factor (MIF), expression of which is induced by hyperoxia in ECs, is increased in PAH [89]. The CD74/MIH complex is involved in PAH and is related to the recruitment of leukocytes to ECs. Indeed, CD74 upregulation is associated with changes in MHCII genes, so this complex contributes to disease progression. It also plays a role in the barrier and proliferation, as it generates hyperproliferation of ECs in advanced PAH [33,90]. Moreover, in a transcriptomic study of different cancer cell types, where lung cancer was included, MIF-CD74 pathways were increased in tumor ECs and related to cellular senescence, whereby cells can secrete bioactive compounds and change their microenvironment [82].

MIF is a cytokine that exists in deposits of alveolar epithelial, alveolar endothelial, and pulmonary macrophages, which is elevated in the serum of patients with ARDS. MIF binds to its cell surface receptor CD74 to activate signaling of several pathways, including p44/p42 MAPK (ERK1/2) and PI3K/AKT, leading to increased survival and decreased p53-dependent apoptosis. CD74 is induced to play its tissue-protective role for the translation of MIF-CD74 signals during hyperoxic injury. Therefore, the MIF-CD74 endothelial signaling pathway may represent a new therapeutic target in lung disease [90].

Although these genes represent promising targets, more research is necessary to confirm these and many other candidates as therapeutic targets in cardiopulmonary disorders.

## 7. Non-Pharmacological Therapeutic Strategies to Protect the Endothelium

The use of drugs as a therapeutic strategy for patients with endothelial dysfunction can be useful; however, they sometimes generate side or harmful effects that worsen the quality of life of patients. This is why the use of other therapeutic strategies could be very beneficial. In this context, studies have shown that both physical exercise and diet have an important impact on the prevention of cardiopulmonary disorders and the recovery of patients.

### 7.1. Exercise

Different studies have shown that moderate physical exercise reduces the incidence of suffering from cardiovascular disease, making it a possible therapeutic strategy for diseases in which oxidative stress plays an important role. In fact, vascular endothelial dysfunction has been linked to a sedentary lifestyle [61,91,92]. Physical training manages to relax the vascular smooth muscles due to a greater production of NO by the ECs and an increase in the expression of endothelial NOS, thus preventing endothelial dysfunction. Moreover, studies carried out in humans and animals have shown that SMCs present in the vessels produce an antioxidant enzyme, superoxide dismutase (SOD), which inhibits ROS, reducing the degradation of NO. Furthermore, there are indications that exercise stimulates the production of NO in the microvasculature, achieving vasodilatory action there and improving myocardial perfusion [61]. Exercise also alters vascular remodeling because it is involved in two processes; (1) it is a powerful stimulator of angiogenesis, which improves blood exchange between different tissues, and (2) it is involved in arteriogenesis, relating to the growth of pre-existent collateral arterioles leading to formation of large conductance arteries that are well capable of compensating for the loss of function of occluded arteries. Therefore, it could be beneficial for those diseases mediated by vascular remodeling [92]. Exercise was initially thought to be harmful for people suffering from respiratory diseases. More recently, it has been reported that patients with PH who exercise have an increase in blood flow velocity as well as in pulmonary perfusion and a decrease in systolic pressure in the pulmonary arteries. It must be taken into account that some patients present arrhythmia after exercising; in these cases, suspension of exercise is recommended. In any case, exercise has positive effects on patients with PH; however, it must always be carried out under the supervision of professionals. Patients with PH who perform regular aerobic exercise and muscle strengthening showed an increase in aerobic capacity in the 6 min walk test, describing that their quality of life had improved. Therefore, an aerobic training routine is an effective therapy in patients with PH [93].

More research is required to further understand the role that exercise plays in these diseases. What is clear is that routine exercise induces an increase in vasodilators produced by the endothelium, a reduction in vascular resistance, and inhibition of platelet aggregation. Therefore, exercise ameliorates the symptoms and prognosis of cardiovascular diseases by reducing patient mortality [61]. In this way, frequent exercise could be considered essential not only as a preventive method for cardiovascular diseases, but also as a possible adjuvant treatment to combat these diseases [92].

### 7.2. Diet

Several studies have shown that a healthy diet based on fruits and vegetables prevents cardiovascular diseases that are closely related to endothelial dysfunction. Various diets have been widely described, such as the Mediterranean diet (with an increased consumption of olive oil as its main characteristic, and also legumes, fruit, vegetables). It has been shown that after consumption of a Mediterranean diet (based on fruits, vegetables, olive oil, and legumes) for 10 years, there was a decrease in CRP, IL-6, IL-7, IL-8, and insulin, lowering blood pressure [94,95]. In a controlled trial that compared the endothelial function in patients with coronary heart disease who consumed a Mediterranean diet or a low-fat diet showed significantly improved endothelial function in those patients who had the Mediterranean diet. The effect of the Mediterranean diet’s beneficial properties is attributed to its fatty acid profile (increased monounsaturated fatty acids (MUFAs) and polyunsaturated fatty acids (PUFAs)) as well as the reduction in carbohydrates compared with other diets. However, the limitations of nutritional intervention studies should be considered [96]. The vegetarian diet (the main characteristic of which is the non-consumption of meat of any kind), with its own variants such as the vegan diet (where no animal-derived products are consumed), have also been studied [97,98]. Even though the increase in the consumption of fruits and vegetables in vegetarian diets is benficial, there have been conflicting results. The lack of consumption of animal-derived foods has sometimes been related to deficiency of some nutrients such as iron, zinc, vitamin D, or vitamin B12, raising awareness of the necessity of dietary supplements for this specific diet [99]. Another diet used is the Dietary Approaches to Stop Hypertension (DASH diet), which is characterized by an increase in the consumption of fish, fruit, and vegetables but most importantly, a reduced consumption of saturated fats, read meats, and alcohol [100]. Interestingly, the DASH diet was developed as a nutritional approach to help patients with arterial hypertension [100].

The diets mentioned above insist on increased consumption of fruit and vegetables. Some of the beneficial components present in fruits and vegetables are antioxidant molecules (vitamin C, vitamin E, and polyphenols) and unsaturated fatty acids. Diets rich in saturated fats are harmful to the endothelium; however, diets rich in PUFAs such as docosapentaenoic acid (DPA) and eicosapentaenoic acid (EPA) are incorporated into the cell membrane, reducing the expression of molecules such as VCAM-1, ICAM-1, and E-selectin. Therefore, they have a beneficial effect on the endothelium. In addition, these acids show anti-inflammatory activity by reducing TNF-α and IL-6 [101,102]. Vitamin C is found in many fruits (such as oranges, grapes, or blueberries) and reduces endothelial dysfunction by increasing NO via stabilizing the eNOS cofactor. Additionally, vitamin E (present in nuts, for example) blocks the production of ROS [103,104,105,106]. Both vitamin C and E have antioxidant roles, which makes their consumption relevant for the improvement of endothelial dysfunction, as it has been previously explained how one of its characteristics is increased oxidative stress on endothelial cells. Fruits are also composed of polyphenols, which contribute to modulating cardiovascular risk factors. One of the fruits richest in polyphenols is the pomegranate. In a study carried out with pigs, it was observed that those who had been fed a diet rich in fats and cholesterol presented endothelial dysfunction, unlike pigs fed with standard feed. Pigs with endothelial dysfunction had a decrease in relaxation capacity induced by acetylcholine and calcium. It was observed in that study that the administration of the Pomanox supplement (pomegranate extract rich in polyphenols) restored vasodilation in the group of animals with a high-fat and cholesterol diet, since this extract activated the Akt/eNOS endothelial axis in the coronary artery, releasing NO and causing vasodilation. In addition, oxidative stress is reduced because activation of this axis reduces the expression of monocyte chemoattractant protein 1 (MCP-1) [107]. Therefore, a good diet is closely related to an improvement in endothelial dysfunction and can be used as a possible therapeutic strategy to combat these diseases.

Lifestyle is very important for the progression of the diseases mentioned in this review. Generally, patients with COPD present malnutrition due to fatigue and early satiety, which are characteristic symptoms of this disease. Adipokines, such as leptin or IL-6, are molecules that participate in the regulation of appetite, and they have been found to be deregulated in patients with COPD, generating an inflammatory process in these patients. This is why energy supplementation is needed. Studies suggest that patients with COPD could benefit from diets rich in vegetables, fruits, soya, fiber, and olive oil, while diets rich in sugars or meats can worsen the disease. In this way, a diet high in lipids and low in carbohydrates improves some of the parameters of lung function, contributing to an improvement in the patient’s quality of life and prognosis. Furthermore, the intake of antioxidants and essential fatty acids is important in order to reduce the inflammatory process and improve the respiratory capacity of patients with COPD [108].

Vitamin B12 helps keep red blood cells oxygenated and prevents megaloblastic anemia, its deficiency being associated with cardiovascular diseases. Studies have shown that B12 has antioxidant and anti-inflammatory properties, being able to regulate the production of inflammatory cytokines, inhibit the production of intracellular peroxide, and prevent cell apoptosis [109]. In fact, in a study carried out by Corcoran et al. in 2009reported that there were differences in B12 levels between patients with ARDS who died and those who survived, with B12 levels being lower in those who died. In addition, it has been reported that patients with ARDS or COVID-19 who were admitted to the ICU also generally presented lower levels of B12 [109,110]. Corcoran’s group observed a positive correlation between B12 and C-reactive protein (CRP), demonstrating the close relationship between this vitamin and the inflammatory response. Furthermore, B12 has antioxidant properties, as it helps maintain intracellular glutathione (GSH) levels, increasing the cytosolic bioavailability of antioxidants. In this way, it reduces ROS and RNS that end up damaging cellular structures [109].

Deficiency in vitamin D (vitD) has previously been correlated with a poor prognosis of PAH. Vitamin D is essential for various processes such as cell growth, oxidative stress, angiogenesis, and metabolism. However, it has been proved that treatment with vitD does not decrease mPAP levels in PAH models, although it did improve endothelial function [111,112].

## 8. Future Perspectives and Conclusions

The endothelium not only provides structure to blood vessels, but also has vital functions for the cardiopulmonary system, such as maintaining the vascular tone, with anti-thrombotic properties, and regulating angiogenesis, among other functions. In the pulmonary system, it is even more relevant, as the endothelium forms a barrier between the air and the blood during gas exchange, being susceptible to multiple changes. As has been seen in this review, there are many cardiopulmonary disorders in which the endothelium is damaged and therefore dysfunctional, generating inflammation, alterations in metabolic pathways, vascular remodeling, and a worse prognosis in general for patients. Each of these hallmarks of endothelial dysfunction has its own signaling pathways that in the end synergize, causing the disease to evolve. Therefore, the presence of a healthy endothelium and maintaining the homeostasis of the air–blood barrier are essential in health and disease prevention. Currently, there are few treatments that can improve the quality of life for patients suffering with cardiopulmonary disorders. Available treatments rather focus on counterbalancing the pathological consequences of endothelial dysfunction to protect the endothelium. For this purpose, new studies are needed to delve deeper into the role of the endothelium in each of these diseases, as it is a potential target for new therapies in cardiopulmonary disorders. Interestingly, it would be very useful to explore new non-pharmacological therapeutic strategies, such as exercise or diet, that suggest providing an improvement in the quality of life of patients and do not involve the intake of drugs. In recent years, development of new techniques such as single-cell RNA sequencing and other omics techniques like transcriptomics has considerably increased the knowledge on the endothelium and its role in both health and pathology. Nevertheless, more research is still needed to further understand and design more specific endothelial targeted therapies and their potential role as therapeutic strategies in cardiopulmonary disorders.

## Figures and Tables

**Table 1:** Epidemiologic data of cardiopulmonary disorders.

Disease	Incidence	Life Expectancy	Symptoms	Detection
Pulmonary Arterial Hypertension (PAH)	1% of the world population [12]	2.8 years without any treatment [13]	Dyspnea and exercise ability impairment [14]	mPAP ≥ 20 mm Hg at rest [15]
Chronic Obstructive Pulmonary Disease (COPD)	10% in people over 40 years old [16]	3.5 years on average [17]	Dyspnea, chronic cough, chest tightness [18]	Spirometry Mild: FEV_1_ ≥ 80% Severe: FEV_1_ < 30% [19]
Acute Respiratory Distress Syndrome (ARDS)	1.5–79 cases per 100,000 [20] COVID-induced ARDS: 32.2% [21]	1 year mortality rate of 11% [22]	Dyspnea, hypoxemia, tachypnea and increased breathing effort [23]	Lung infiltrates in chest CT PaO_2_/FiO_2_ < 300 mm Hg PEEP > 5 cm H_2_O [23]
Obstructive Sleep Apnea (OSA)	9% to 38%, higher prevalence in men and in elder groups [24]	Dependent on comorbidities	Tiredness, insomnia, morning headaches [25]	Apnea hypopnea index (AHI) [25]

FEV_1_: Expiratory volume in one second; mPAP: Mean pulmonary arterial pressure. PaO_2_/FiO_2_: O_2_ partial arterial pressure (PaO_2_) to the inspired oxygen fraction (FiO_2_) ratio. PEEP: positive end-expiratory pressure.

**Table 2:** Biomarkers of endothelial damage.

Disease	Biomarkers
Pulmonary Hypertension (PH)	ET-1, Ang-2, ADMA, IL-6, IL-8, IL-10, IL-12p70, BNP, vWF
Obstructive Sleep Apnea (OSA)	HbA1c, CRP, EPO, IL-6, IL-10, VCAM1, NO, HIF-1α, NF-kß, TNF-α
Chronic Obstructive Pulmonary Disease (EPOC)	IL-6, CRP, fibrinogen, CC16, sRAGE
Acute Respiratory Distress Syndrome (ARDS)	CRP, IL-1β, TNF-α, IL-8, TGFβ1

ADMA: asymmetric dimethylarginine; Ang: angiopoietin; BNP: brain natriuretic peptide; CC16: club cell protein 16; EPO: erythropoietin; ET-1: endothelin1; HbA1c: glycated hemoglobin A; HIF-1α: hypoxia-inducible factor 1-alpha; IL: interleukin; NF-kß: Nuclear factor kappa-light-chain-enhancer of activated B cells; NO: nitrogen oxide; CRP: C-reactive protein; Srage: soluble receptor for advanced glycation end products; TNF-α: tumor necrosis factor; VCAM1: vascular cell adhesion protein 1; vWF: von Willebrand factor [57].

## Data Availability

No new data were created or analyzed in this study. Data sharing is not applicable to this article.
